# Inhibition of deubiquitination by PR‐619 induces apoptosis and autophagy via ubi‐protein aggregation‐activated ER stress in oesophageal squamous cell carcinoma

**DOI:** 10.1111/cpr.12919

**Published:** 2020-10-31

**Authors:** Longhao Wang, Miaomiao Li, Beibei Sha, Xuanyu Hu, Yaxin Sun, Mingda Zhu, Yan Xu, Pingping Li, Yating Wang, Yanyan Guo, Jiangfeng Li, Jianxiang Shi, Pei Li, Tao Hu, Ping Chen

**Affiliations:** ^1^ Academy of Medical Sciences School of Basic Medical Sciences Zhengzhou University Zhengzhou China; ^2^ Precision Medicine Center Henan Institute of Medical and Pharmaceutical Sciences & BGI College Zhengzhou University Zhengzhou China; ^3^ Henan Key Laboratory of Precision Clinical Pharmacy The First Affiliated Hospital of Zhengzhou University Zhengzhou China

**Keywords:** apoptosis, autophagy, ER stress, oesophageal squamous cell carcinoma, PR‐619

## Abstract

**Objectives:**

Targeting the deubiquitinases (DUBs) has become a promising avenue for anti‐cancer drug development. However, the effect and mechanism of pan‐DUB inhibitor, PR‐619, on oesophageal squamous cell carcinoma (ESCC) cells remain to be investigated.

**Materials and Methods:**

The effect of PR‐619 on ESCC cell growth and cell cycle was evaluated by CCK‐8 and PI staining. Annexin V‐FITC/PI double staining was performed to detect apoptosis. LC3 immunofluorescence and acridine orange staining were applied to examine autophagy. Intercellular Ca^2+^ concentration was monitored by Fluo‐3AM fluorescence. The accumulation of ubi‐proteins and the expression of the endoplasmic reticulum (ER) stress‐related protein and CaMKKβ‐AMPK signalling were determined by immunoblotting.

**Results:**

PR‐619 could inhibit ESCC cell growth and induce G2/M cell cycle arrest by downregulating cyclin B1 and upregulating p21. Meanwhile, PR‐619 led to the accumulation of ubiquitylated proteins, induced ER stress and triggered apoptosis by the ATF4‐Noxa axis. Moreover, the ER stress increased cytoplasmic Ca^2+^ and then stimulated autophagy through Ca^2+^‐CaMKKβ‐AMPK signalling pathway. Ubiquitin E1 inhibitor, PYR‐41, could reduce the accumulation of ubi‐proteins and alleviate ER stress, G2/M cell cycle arrest, apoptosis and autophagy in PR‐619‐treated ESCC cells. Furthermore, blocking autophagy by chloroquine or bafilomycin A1 enhanced the cell growth inhibition effect and apoptosis induced by PR‐619.

**Conclusions:**

Our findings reveal an unrecognized mechanism for the cytotoxic effects of general DUBs inhibitor (PR‐619) and imply that targeting DUBs may be a potential anti‐ESCC strategy.

## Graphical abstract

PR‐619 could inhibit oesophageal squamous cell carcinoma cell growth and induce G2/M cell cycle arrest by downregulating the expression of cyclin B1 and upregulating the protein level of p21. Meanwhile, PR‐619 treatment led to the accumulation of ubiquitylated proteins, induced ER stress and triggered apoptosis by ATF4‐Noxa axis. Moreover, the ER stress increased cytoplasmic Ca^2+^ and then stimulated autophagy through Ca^2+^‐CaMKKβ‐AMPK signaling pathway.

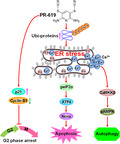

## 1 INTRODUCTION

Oesophageal cancer is one of the common malignancies with high mortality rates in the world.^1^, ^2^ Histologically, oesophageal squamous cell carcinoma (ESCC) is the primary subtype in Asia, including China. Although there are rapid developments in diagnosis and therapies, the prognosis of oesophageal cancer patients is still abysmal, and the 5‐year relative survival rates are only 30% in China^1^ and 19% worldwide.^2^ Therefore, it is crucial to investigate the mechanism of cancer progression and find novel candidates for targeted therapeutic strategies.

Deubiquitinases (DUBs) are essential components of the ubiquitin‐proteasome system (UPS). By hydrolysing the isopeptide bond that present at the C‐terminus of ubiquitin molecule, DUBs remove ubiquitin chains, regulate the activation and localization of target proteins and thus participate in the regulation of various cellular processes including apoptosis, senescence and autophagy.^3^, ^4^ Recent reports showed that many DUBs overexpressed in solid tumours, leukaemias and myelomas.^5^, ^6^, ^7^, ^8^ USP14 overexpressed in epithelial ovarian cancer,^9^ colorectal cancer,^10^ lung cancer^11^ and oesophageal cancer,^12^, ^13^ and it was closely related to more inferior overall survival rate and prognosis. The increased expression of USP7/HAUSP was also reported to participate in the development of multiple cancers, such as multiple myeloma,^14^ oesophageal cancer,^15^ gliomas ^16^ and ovarian cancer.^17^, ^18^ The relationship between abnormally activated or expressed of DUBs and cancers was reviewed in detail in several recent papers.^5^, ^6^, ^7^, ^8^ These reviews highlight that DUBs represent novel candidate targets for drug development.

Inactivation of DUBs by inhibitors showed promising antitumour activity in multiple tumours. Chauhan *et al* ^14^ discovered that P5091 was a specific inhibitor of deubiquitylating enzyme USP7. P5091 significantly inhibited cell growth of ovarian cancer,^19^ oesophageal cancer^15^ and colorectal cancer^20^ in vitro and in vivo. Meanwhile, P5091 induced apoptosis in multiple myeloma cells resistant to conventional and Bortezomib therapies.^14^ D´Arcy *et al* first reported b‐AP15 and described it as an inhibitor of USP14 and UCH37/UCHL5.^21^ And then, b‐AP15 was identified as an anti‐cancer deubiquitinase inhibitor in many cancers.^13^, ^22^, ^23^, ^24^ Using activity‐based chemical proteomics, Altun *et al* characterized the small molecule PR‐619 as a broad‐range DUB inhibitor.^25^ PR‐619 treatment led to the striking accumulation of poly‐ubiquitinated proteins and components of the 26S proteasome complex without direct impairment of proteasomal proteolysis.^25^ Subsequently, PR‐619 was widely used to investigate the role of ubiquitination in various cell and physiological processes. PR‐619 participated in the trafficking of Ca^2+^‐activated K^+^ channel (KCa3.1),^26^ dynein localization during mitosis,^27^ oocytes mature^28^ and HIV‐1 replication.^29^ PR‐619 affected the microtubule network and caused protein aggregation in neural cells.^30^ Administration of PR‐619 attenuated renal fibrosis in vitro and in vivo by reducing Smad4 expression.^31^ PR‐619 induced autophagy in oligodendroglia cells^32^ and sensitized normal human fibroblasts to TRAIL‐mediated cell death.^33^ More recently, Kuo *et al* reported that PR‐619 could effectively induce dose‐ and time‐dependent cytotoxicity and ER stress‐related apoptosis in metastatic bladder urothelial carcinoma (UC) and potentiate cisplatin‐induced cytotoxicity in UC.^34^ However, little is known about the effects and mechanism of PR‐619 on oesophageal cancer cells.

Here, we found that PR‐619 treatment inhibited oesophageal squamous cell carcinoma cell growth and led to G2/M cell cycle arrest by reducing the expression of cyclin B1 and upregulating the protein level of p21. Meanwhile, PR‐619 treatment induced accumulation of ubiquitinated proteins that could cause ER stress and triggered apoptosis by the ATF4‐Noxa axis. Moreover, the ER stress increased the level of cellular Ca^2+^ concentration and then stimulated protective autophagy through Ca^2+^‐CaMKKβ‐AMPK pathway. CaMKKβ inhibitor STO‐609 and AMPK inhibitor Compound C (CC) could inactivate AMPK and attenuate the formation of autophagy in ESCC cells. Ubiquitin E1 inhibitor, PYR‐41, could reduce the accumulation of ubiquitinated proteins and alleviate ER stress, G2/M cell cycle arrest, apoptosis and autophagy. Furthermore, blocking autophagy with chloroquine (CQ) or bafilomycin A1 (BafA1) enhanced the cell growth inhibition and apoptotic effect of PR‐619 in ESCC cell lines. These findings reveal an unrecognized mechanism for the cytotoxic effects of general DUBs inhibitor (PR‐619) and indicate that targeting DUBs may be a potential anti‐ESCC strategy.

## 2 MATERIALS AND METHODS

### 2.1 Cell culture and regents

Human oesophageal squamous cell carcinoma cell line Kyse30, Kyse450, EC1 and EC109 were cultured in DMEM (BI) medium containing 10% FBS (BI) at 37℃ with 5% CO_2_. PR‐619 (a pan‐DUB inhibitor), STO‐609 (a CaMKK inhibitor), Compound C (CC) (an AMPK inhibitor) and PYR‐41 (a ubiquitin E1 inhibitor) were purchased from MedChemExpress (MCE) and dissolved in dimethyl sulfoxide (DMSO). Chloroquine (CQ) was purchased from Sigma‐Aldrich and was dissolved in phosphate‐buffered saline (PBS). Bafilomycin A1(BafA1) was purchased from Sigma‐Aldrich and dissolved in DMSO.

### 2.2 Cell viability and colony assay

ESCC cell lines Kyse30, Kyse450, EC1 and EC109 were seeded into 96‐well plates and treated with PR‐619 or DMSO (0.1%) for 48 hours. Cell viability was detected using the Cell Counting Kit‐8 (CCK‐8) kit (Beyotime Institute of Biotechnology, China). Cell growth was also examined by colony formation assay. Five hundred cells were seeded into 6‐well plates in triplicate, treated with DMSO (0.1%) or PR‐619 and then incubated for 10 days. The colonies were fixed with 4% paraformaldehyde (Solarbio, China) and stained with crystal violet (Beyotime, China). Colonies comprising 50 cells or more were counted as previously described.^35^

### 2.3 Cell cycle analysis

Kyse30 and Kyse450 cells were treated with DMSO (0.1%) or PR‐619 for 24 hours, respectively. Cells were collected, fixed with 70% alcohol, stained with propidium iodide (PI) solution (50 μg/mL) (Solarbio, China) containing 50 μg/mL RNase A (Solarbio, China) at 37˚C for 30 minutes and detected by flow cytometry (Becton Dickinson FACScan; Becton‐Dickinson, San Jose, CA, USA). Each phase distribution was analysed by ModFit LT 3.1 software (Verity Software House, Inc, Topsham, ME, USA).

### 2.4 Detection of apoptosis and the activity of caspase3 (CASP3)

Kyse30 and Kyse450 cells were treated with DMSO (0.1%) or PR‐619 for 48 hours, respectively. Apoptosis was determined by Annexin V/Fluorescein Isothiocyanate (FITC)/PI Apoptosis kit (Tianjin Sungene Biotech Co., Ltd., China), and Annexin V^+^ cells were collected as apoptotic cells. The activity of CASP3 was measured by the CaspGLOW Fluorescein Active Caspase3 Staining kit (BioVision, Inc, Milpitas, CA, USA) according to the manufacturer's instruction.

### 2.5 Evaluation of mitochondrial membrane depolarization

Kyse30 and Kyse450 cells were treated with DMSO or PR‐619 for 24 hours, respectively. Mitochondrial membrane depolarization was detected with the mitochondrial membrane potential assay kit with JC‐1 according to the manufacturer's protocol (Yeasen Inc, China). The data were acquired and analysed by flow cytometry. Cells with intact mitochondria displayed high red fluorescence and appeared in the upper right quadrant of the scatterplots. In contrast, cells that had lost mitochondrial membrane potential (MMP) appeared in the right lower quadrant displaying highlighted green and low red fluorescence.

### 2.6 Western blotting assay

Kyse30 and Kyse450 cells were treated with DMSO or PR‐619d for 48 hours, respectively. Cell lysates were prepared for Western blotting analysis, with antibodies against p21, p‐WEE1 (Ser642), WEE1, Cyclin B1, p‐Histone H3 (Ser10), Cdc25c, ATF4, BiP, p‐ACC(Ser79), ACC, LC3, p‐eIF2α (Ser51), eIF2α, FOXO3a, cleaved CASP9, cleaved CASP3, cleaved PARP, PARP, Bax, Bak, Bcl‐xl, Mcl‐1, XIAP, CIAP1, CIAP2 (Cell Signaling Technology, Inc, Boston, MA), Noxa (Millipore, Billerica, MA), c‐Myc and Ub (Santa Cruz Biotechnology, Santa Cruz, CA), HIF‐1α and AMPKα (Abgent, Shanghai, China), p‐AMPKα (Thr172), Histone H3, Capase3 and Capase9 (Beyotime, China). GAPDH, Tubulin and β‐actin were used as loading controls. All the primary antibodies were diluted in 1:1000 and incubated at 4˚C overnight. After washed with Tris‐Buffered Saline Tween‐20 (TBST), the membrane was incubated with secondary antibodies peroxidase‐conjugated goat anti‐mouse IgG or peroxidase‐conjugated goat anti‐rabbit IgG (diluted in 1:3000, ZGSB‐Bio, Inc, China) for 2 hours at room temperature. Then, the membrane was detected using an ECL Kit (Beyotime, China).

### 2.7 Gene silencing using siRNA

Kyse30 and Kyse450 cells were transfected with siRNA oligonucleotides (final concentration: 100 nmol/L) synthesized by GenePharma (Shanghai GenePharma Co. Ltd, China) using Lipofectamine 8000 (Beyotime, China). The sequences of the siRNA were as follows: siNoxa: GUAAUUAUUGACACAUUUC^35^; siATF4: GCCUAGGUCUCUUAGAUGA^35^; siControl: UUCUCCGAACGUGUCACGU.

### 2.8 Acridine orange (AO) staining for autophagy detection

Acridine orange staining was performed to detect autophagy according to published protocol.^36^, ^37^ Briefly, cells were treated with DMSO or PR‐619 (8 µmol/L) for 24 hours and stained with acridine orange (Solarbio, China) in PBS containing 5% FBS at 37°C for 30 minutes. Cells were washed and observed under fluorescence microscopy (magnification: 200×; Nikon, Nikon Inc, Tokyo, Japan). The formation of acidic vesicular organelles (AVOs) was examined under fluorescence microscopy. AVOs, such as autolysosomes, were orange/red. Non‐AVO areas (cytoplasm, nucleus, and nucleolus) were green.

### 2.9 LC3 Immunofluorescence staining

Kyse30 and Kyse450 cells were plated on a glass‐bottom cell culture dish and treated with 0.1% DMSO or PR‐619 (8 µmol/L) for 24 hours. Cells were fixed with methanol at −20°C for 30 minutes, blocked by 5% BSA and then incubated with LC3 primary antibody (1:200, overnight at 4℃) (Cell Signaling Technology, Inc, Boston, MA) and Alexa Fluor^®^ 488 Goat Anti‐Rabbit IgG (H + L) secondary antibody (green) (1:500, 2 hours at room temperature) (Beyotime, China), respectively. The nuclei were stained by DAPI (blue) (5 μg/mL, 20 minutes at room temperature) (Beyotime, China). Images were captured using fluorescence microscopy (magnification: 200×; Nikon, Nikon Inc, Tokyo, Japan).

### 2.10 Measurement of intracellular Ca^2+^

The levels of intracellular Ca^2+^ were detected using the Fluo‐3 AM fluorescence working solution (Beyotime, China) as the instruction described. In brief, Kyse30 and Kyse450 cells were treated with DMSO or PR‐619 for 24 hours, and Fluo‐3 AM fluorescence working solution was added to cells at 37℃ for another 30 minutes. Cells were washed three times with PBS buffer, and pictures were captured using fluorescence microscopy (magnification: 200×; Nikon, Nikon Inc, Tokyo, Japan). Intercellular Ca^2+^ concentration was monitored by flow cytometry.

### 2.11 Statistical analysis

The statistical significance of differences between groups was assessed using GraphPad Prism5 software. (GraphPad Software, Inc, La Jolla, CA, USA). The *t* test was used for the comparison of parameters between groups. For all tests, * represented the difference between the two groups was significant.

## 3 RESULTS

### 3.1 PR‐619 suppressed the growth of ESCC cells

**FIGURE 1:**
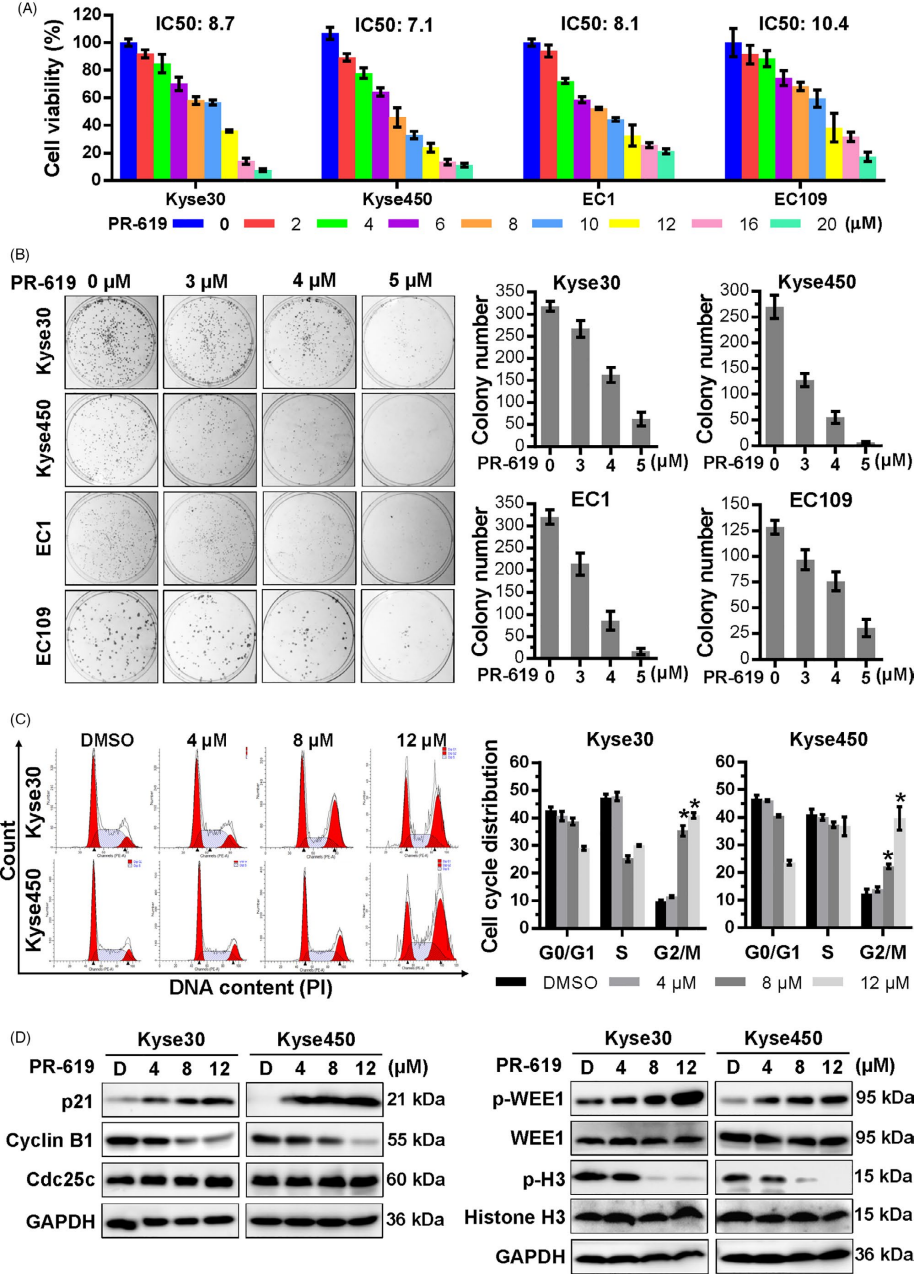
PR‐619 inhibited oesophageal cancer cell growth, colony formation and induced cell cycle arrest. A, Effect of PR‐619 on the viability of four ESCC cells, Kyse450, Kyse30, EC1 and EC109. Cells were treated with 0.1% DMSO or PR‐619 for 48 h, and viability was detected with the CCK‐8 kit. IC50 was calculated by SPSS software (version). B, Efficacy of PR‐619 on colony formation of ESCC cells. ESCC cells were treated with PR‐619 at different concentrations for 10 d and then fixed, stained and counted as described in the Materials and Methods section. Colons were captured as shown in the left panel, and colony number was statistically analysed, as shown in the right panel. C, PR‐619 triggered G2/M cell cycle arrest in oesophageal cancer cells. Kyse30 and Kyse450 cells were treated with PR‐619 at different concentrations for 24 h, followed by PI staining and FACS analysis for cell cycle profile (left panel). Distribution was analysed by Modifit and GraphPad software (right panel). D, Effect of PR‐619 on the expression of cell cycle‐related proteins. Kyse30 and Kyse450 cells were treated with PR‐619 at indicated concentrations and subjected to Western blotting subsequently. GAPDH was used as the loading control. All data were representative of at least three independent experiments (n = 3; error bar, SD)

As suggested, PR‐619 inhibited the majority of DUBs at a concentration of 5‐20 μmol/L.^25^ So, we examined the anti‐ESCC efficacy of PR‐619 at a concentration of 2‐20 μmol/L. Results showed that PR‐619 inhibited the growth of ESCC cells Kyse30, Kyse450, EC1 and EC109, as evidenced by dose‐dependent inhibition of cell proliferation (Figure 1A) and colony formation (Figure 1B).

### 3.2 PR‐619 induced G2 phase cell cycle arrest in oesophageal cancer cells

To elucidate the growth suppression mechanism of PR‐619, the cell cycle profile was examined after treatment with PR‐619. As shown in Figure 1C, treatment with PR‐619 induced G2/M cell cycle arrest in a dose‐dependent manner. The expression of cell cycle‐related proteins was further examined by Western blotting. Results showed that PR‐619 induced the phosphorylation of Wee1 (inhibitor of G2‑M phase transition^38^, ^39^), increased the protein level of p21 and decreased the expression of cyclin B1 and p‐Histone H3, a hallmark of M‐phase cells^39^, ^40^ (Figure 1D), while PR‐619 treatment has no obvious effect on the expression of Cdc25c (an essential regulator of G2/M transition^41^, ^42^) and the total protein level of Wee1 and Histone H3 (Figure 1D). Take it together, PR‐619 treatment led to G2 phase cell cycle arrest.

### 3.3 Noxa played a vital role in PR‐619‐induced intrinsic apoptosis

**FIGURE 2:**
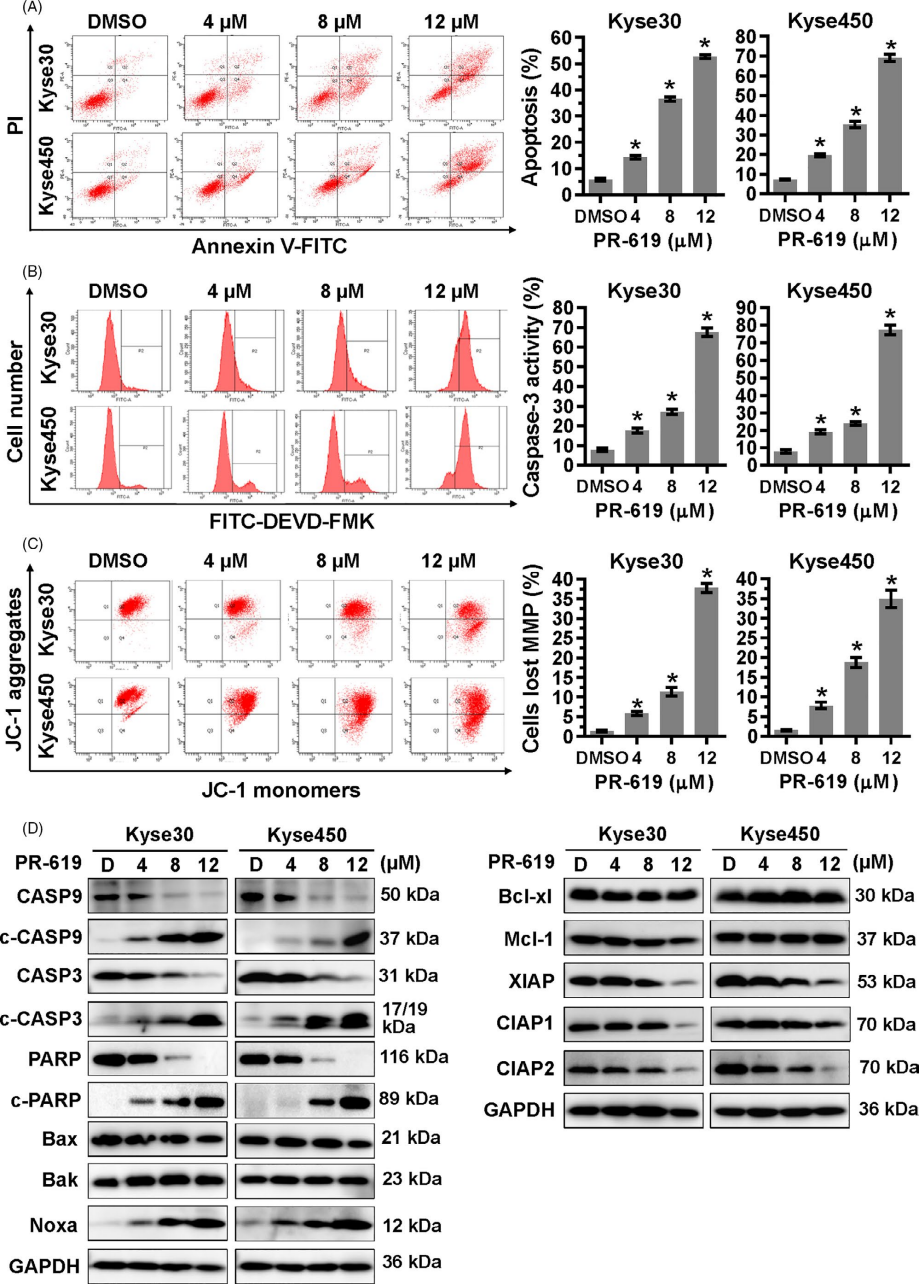
PR‐619 induced intrinsic apoptosis. Kyse30 and Kyse450 cells were treated with PR‐619 for 48 h. A, Apoptosis was determined by FACS analysis using Annexin V‐FITC/PI double‐staining kit (left panel), and Annexin V^+^ cell populations were defined as apoptosis (right panel). B, CASP3 activity was also analysed with FACS (left panel). The percentage of cells with active caspase3 was shown in the right panel. C, PR‐619 induced mitochondrial membrane depolarization. Cells were treated with PR‐619 and analysed by FACS as described in Materials and Methods section. D, Effect of PR‐619 on the expression of apoptotic proteins, pro‐apoptotic and anti‐apoptotic proteins. Kyse30 and Kyse450 cells were treated with PR‐619 for 48 h, and cell extracts were prepared for Western blotting analysis. GAPDH was used as the loading control. All data were representative of at least three independent experiments (n = 3; error bar, SD)

Next, we examined whether apoptosis was also responsible for the growth inhibition effect of PR‐619. Results showed that PR‐619 treatment triggered apoptosis and significantly increased the Annexin V^+^ cells (Figure 2A) and caspase‐3 (CASP3)‐activated cells (Figure 2B). Besides, PR‐619 effectively induced the cleavage of CASP9, CASP3 and PARP (Figure 2D). These results suggested that PR‐619 triggered apoptosis in ESCC cells.

**FIGURE 3:**
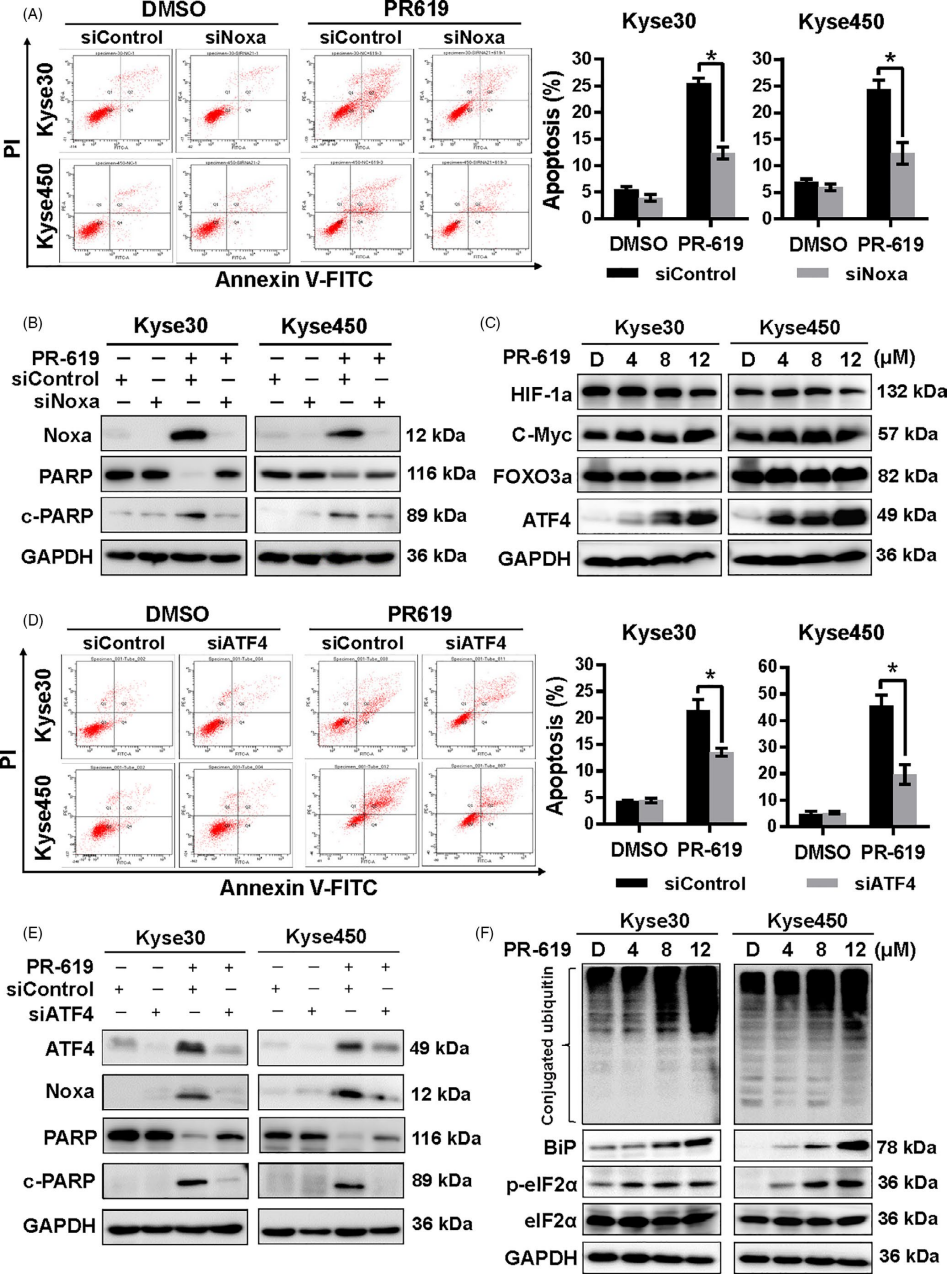
ATF4‐Noxa was responsible for PR‐619‐induced apoptosis in ESCC cells. A, Noxa was critical for apoptosis induced by PR‐619 in ESCC cells. After transfected with the control siRNA or Noxa siRNA, Kyse30 and Kyse450 cells were further treated with PR‐619 (8 μΜ) for 48 h. Apoptosis was quantified by Annexin V‐FITC/PI double‐staining analysis (left panel), and Annexin V^+^ cell populations were defined as apoptosis (right panel). B, Efficiency of siNoxa and its effect on the level of cleaved PARP. Kyse30 and Kyse450 cells were treated as described in panel A, and cell proteins were extracted for Western blotting analysis. GAPDH was used as the loading control. C, Screen of Noxa‐related transcription factors. Kyse30 and Kyse450 cells were treated with DMSO or PR‐619, and cell lysates were measured by Western blotting with specific antibodies. GAPDH was used as the loading control. D, The expression of ATF4 was responsible for PR‐619‐induced apoptosis in ESCC cells. After transfected with the control siRNA or ATF4 siRNA, Kyse30 and Kyse450 cells were further treated with PR‐619 (8 μΜ) for 48 h. Apoptosis was examined by Annexin V‐FITC/PI double‐staining analysis (left panel), and Annexin V^+^ cell populations were defined as apoptosis (right panel). E, Knockdown efficiency of siATF4 and its effect on the expression of Noxa and cleaved PARP were measured by Western blotting analysis. GAPDH was used as the loading control. F, PR‐619 treatment accumulated ubiquitinated proteins and activated ER stress. Kyse30 and Kyse450 cells were treated with PR‐619, and cell lysates were assessed by Western blotting with specific antibodies. GAPDH was used as the loading control. All data were representative of at least three independent experiments (n = 3; error bar, SD)

Based on the results presented above, the change of mitochondrial membrane potential (MMP), another classical marker of the activation of intrinsic apoptosis, was also examined. Results showed that PR‐619 treatment led to the loss of MMP (Figure 2C), which further indicated the induction of intrinsic apoptosis. Therefore, the expression of BCL‐2 family members, including pro‐apoptotic proteins (Noxa, Bak and Bax) and anti‐apoptotic proteins (Bcl‐xl, Mcl‐1, CIAP1, CIAP2 and XIAP), was examined in Kyse30 and Kyse450 cells after PR‐619 treatment (Figure 2D). Among these proteins, the pro‐apoptotic protein Noxa was significantly induced (Figure 2D). Moreover, the downregulation of Noxa via siRNA silencing remarkably suppressed PR‐619‐induced apoptosis and resulted in the decrease of Annexin V^+^ cells (Figure 3A) and cleaved PARP (Figure 3B). These findings highlighted a pivotal role of Noxa in PR‐619‐induced intrinsic apoptosis.

### 3.4 PR‐619 triggered ER stress and activates ATF4‐Noxa Axis to induce intrinsic apoptosis

Noxa is a crucial mediator of chemotherapy‐induced apoptosis, which is transactivated by several transcription factors (TFs).^43^ Firstly, we examined the expression level of Noxa regulation‐related TFs after PR‐619 treatment. Results showed that PR‐619 treatment significantly increased the expression of ATF4 and slightly decreased the expression of FOXO3a, while other TFs changed slightly (Figure 3C). Furthermore, knockdown of ATF4 rescued PR‐619‐induced apoptosis (Figure 3D), downregulated the expression of Noxa and led to a lower level of c‐PARP (Figure 3E). ATF4 is an essential factor in ER stress, so the expression of ER stress‐related proteins was examined. Results showed that PR‐619 treatment induced the accumulation of poly‐ubiquitinated proteins (Figure 3F) and increased the expression of ER stress‐related proteins, including BiP and p‐eIF2α (Figure 3F). These results indicated that PR‐619 triggered ER stress and induced ATF4‐Noxa‐mediated apoptosis in ESCC cells.

### 3.5 PR‐619 induced pro‐survival autophagy

**FIGURE 4:**
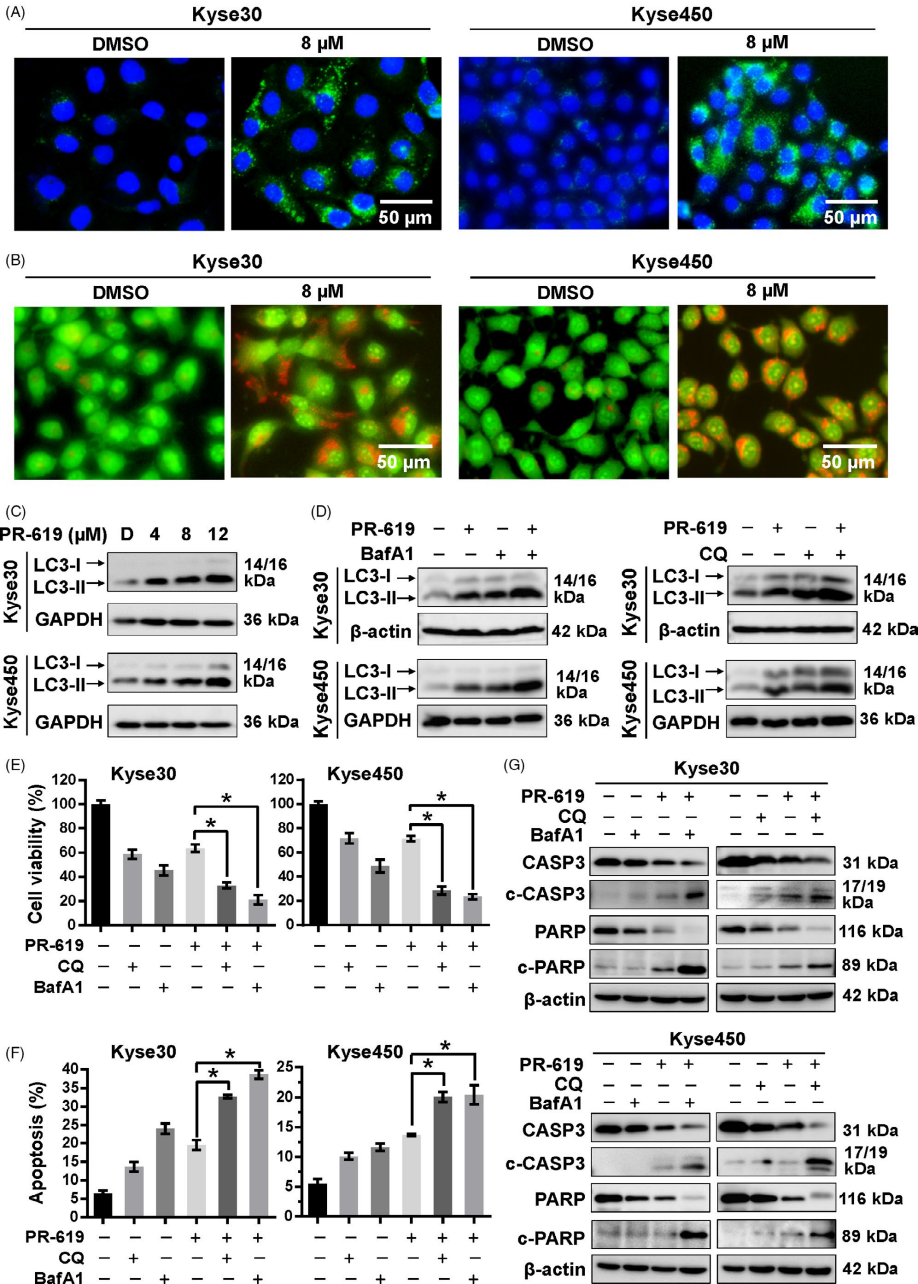
PR‐619 treatment activated autophagy. A, Immunofluorescence of LC3. Kyse30 and Kyse450 cells were treated with PR‐619 (8 μΜ) for 24 h. Cells were then incubated with LC3 primary antibody (1:200, overnight at 4℃) and Alexa Fluor^®^ 488 Goat Anti‐Rabbit IgG (H + L) secondary antibody (green) (1:500, 2 h at room temperature), respectively. The nuclei were stained by DAPI (blue) (5 μg/mL, 20 min at room temperature). Images were captured using fluorescence microscopy (magnification: 200×). Representative images were shown. B, AO staining after PR‐619 treatment. Kyse30 and Kyse450 cells were treated with PR‐619 for 24 h and then stained with AO. The formation of acidic vesicular organelles (AVOs) was examined under fluorescence microscopy. AVOs, such as autolysosomes, were orange/red. Non‐AVO areas (cytoplasm, nucleus, and nucleolus) were green. C, Detection of the expression of LC3. Kyse30 and Kyse450 cells were treated with PR‐619, and cell extracts were prepared for Western blotting analysis. GAPDH was used as the loading control. D, Treatment with CQ or BafA1 suppress LC3‐II degradation. Kyse30 and Kyse450 cells were treated with 8 μmol/L PR‐619 alone or in combination with CQ (12 μmol/L) or BafA1 (8 nmol/L). Cell lysates were analysed by immunoblotting with antibody against LC3. Tubulin was used as the loading control. E, Blockage of autophagy enhances PR‐619‐induced cell growth suppression of oesophageal cancer cell. Kyse30 and Kyse450 cells were treated with 8 μmol/L PR‐619 alone or in combination with CQ (8 μmol/L) or BafA1 (3 nmol/L). Cell viability was measured using the CCK‐8 assay. F and G, Blockage of the autophagic response increased PR‐619‐induced apoptosis of oesophageal cancer cells. Kyse30 and Kyse450 cells were treated with 8 μmol/L PR‐619 alone or in combination with CQ (12 μmol/L) or BafA1 (8 nmol/L). Apoptosis was determined by the Annexin V‐FITC/PI double‐staining analysis (F). Total PARP and caspase 3 and cleaved PARP and caspase 3 were detected by immunoblotting (G). β‐actin was used as the loading control. All data were representative of at least three independent experiments (n = 3; error bar, SD)

Previous reports showed that DUBs were involved in the regulation of autophagy,^44^ so we investigated whether broad‐range DUB inhibitor PR‐619 also could active autophagy in ESCC cell lines. As shown in Figure 4, PR‐619 treatment induced autophagy, evidenced by LC3 immunofluorescence staining (Figure 4A), AO staining (Figure 4B) and the increased expression of LC3‐II (Figure 4C). Moreover, the expression of LC3‐II was further significantly elevated upon PR‐619 co‐treatment with the autophagy inhibitor, both CQ and BafA1 (Figure 4D), indicating that CQ or BafA1 potently blocked the late steps of autophagic flux induced by PR‐619.

Furthermore, we found that the inhibition of autophagy with either CQ or BafA1 significantly enhanced PR‐619‐induced inhibition of cell viability (Figure 4E) and PR‐619‐induced apoptosis (Figure 4F). Co‐treatment with CQ or BafA1 resulted in an increased level of cleaved PARP and caspase3 than treatment with PR‐619 alone (Figure 4G) in both Kyse30 and Kyse450 cells. These results revealed that PR‐619 activated pro‐survival autophagy, and blockage of the autophagic response significantly enhanced the cell growth inhibition efficacy of PR‐619 on ESCC cells.

### 3.6 PR‐619 induced autophagy through activation of Ca^2+^‐CaMKKβ‐AMPK signalling cascade

**FIGURE 5:**
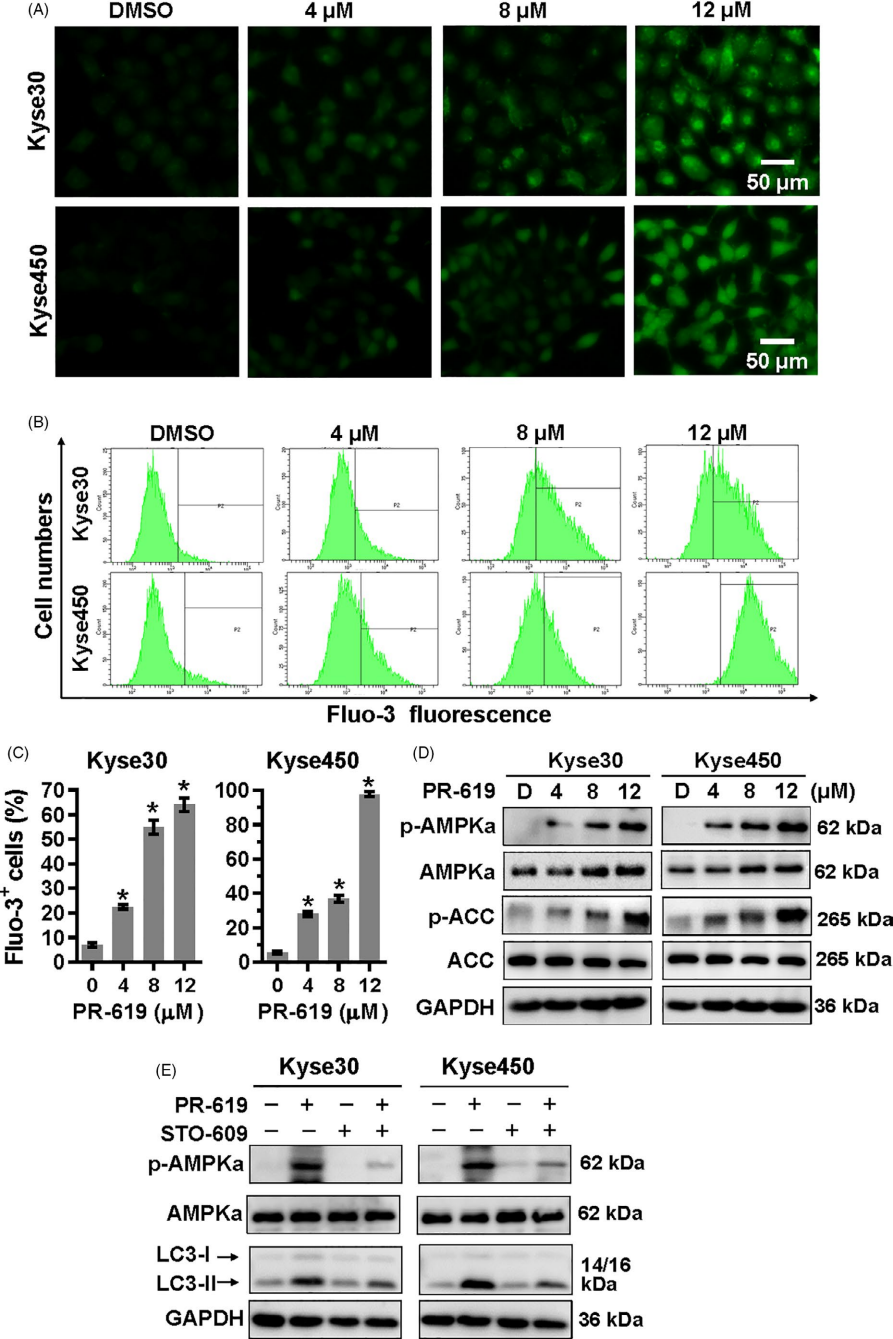
Ca^2+^‐CaMKKβ signalling in PR‐619 treatment cells was involved in AMPKα activation. A to C, Detection of intracellular Ca^2+^. Kyse30 and Kyse450 cells were treated with PR‐619 as indicated. Twenty‐four h later, cells were collected and washed with PBS. Then, Fluo‐3AM was added into the cells as described in the instruction and incubated for 30 min at 37℃. Cells were washed with PBS again and pictures were captured under a microscope. Represented pictures were shown in panel A. Fluo‐3^+^ cells were detected (B) and statistically analysed (C) using FACS. D, PR‐619 treatment activated AMPKα. Kyse30 and Kyse450 cells were treated with PR‐619, as indicated. Cells were collected, and proteins were extracted and analysed by Western blotting with specific antibodies. GAPDH was used as the loading control. E, STO‐609 inhibited the activation of AMPKα and rescued autophagy in PR‐619‐treated cells. Kyse30 and Kyse450 cells were treated with PR‐619 single (8 µmol/L) or combined with STO‐609 (10 µmol/L). Then, proteins were extracted and analysed using specific antibodies against AMPKα, p‐AMPKα and LC3. GAPDH was used as the loading control. All data were representative of at least three independent experiments (n = 3; error bar, SD)

It has been well documented that ER stress can disrupt Ca^2+^ homeostasis inside the ER and lead to Ca^2+^ releasing into other cellular compartments.^45^ Accumulating evidence also shows that cytosolic calcium is a potent inducer of autophagy by CaMKKβ‐AMPK signalling pathway.^45^, ^46^, ^47^ Therefore, we next investigate whether PR‐619 induced autophagy through the activation of Ca^2+^‐CaMKKβ‐AMPK signalling cascade. The microscopy images showed that PR‐619 treatment increased intracellular Ca^2+^ concentration in a dose‐dependent manner (Figure 5A). The fluorescence intensity of the Ca^2+^ dye and the number of Fluo‐3^+^ cells also gradually increased in response to PR‐619 (Figure 5B‐C). Meanwhile, PR‐619 treatment significantly upregulated the protein level of p‐AMPKα and p‐ACC, a substrate of AMPK (Figure 5D). Furthermore, CaMKKβ inhibitor, STO‐609, effectively downregulated the expression of p‐AMPKα and LC3II (Figure 5E). Collectively, these findings suggest that the increase cellular Ca^2+^ and activity of Ca^2+^‐CaMKKβ signalling in PR‐619 treatment cells are implicated in AMPK activation, which perhaps is involved in autophagy.

**FIGURE 6:**
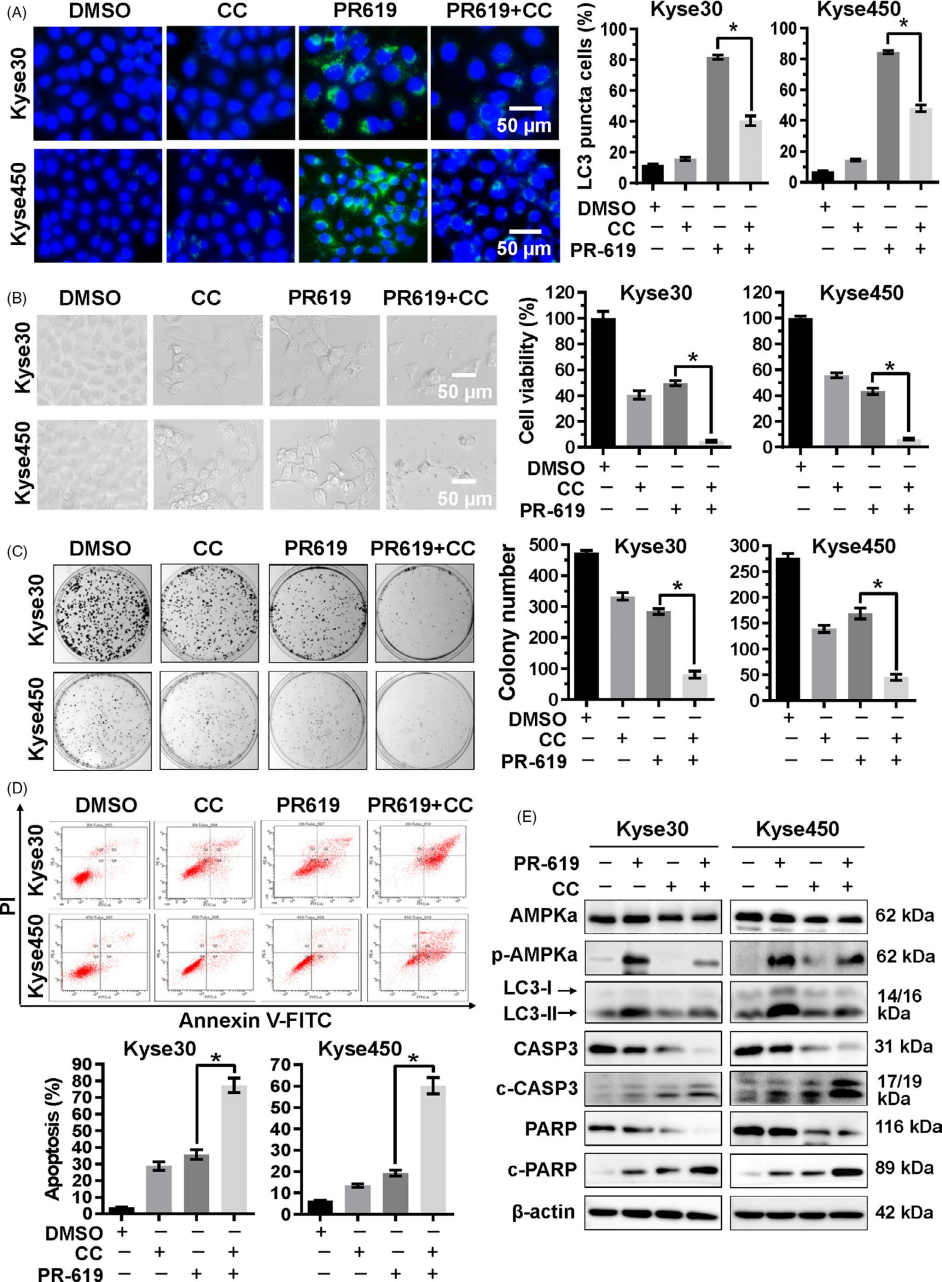
AMPK signalling pathway was involved in the PR‐619‐induced autophagy and apoptosis. A, Blockage of AMPK reduced PR‐619 induced autophagy. Kyse30 and Kyse450 cells were treated with PR‐619 single (8 µmol/L) or combined with CC (8 µmol/L), an AMPK inhibitor. LC3 was detected using immunofluorescence assay as described in material and methods, reprehensive pictures were captured (left panel), and LC3 puncta cells were statistically analysed (right panel). B, Blockage of AMPK enhanced the inhibition of cell viability in PR‐619‐treated cells. Kyse30 and Kyse450 cells were treated with PR‐619 single (8 µmol/L) or combined with CC (4 µmol/L) as described above. Cell proliferation was observed and captured under inverted microscope (left panel). Cell viability was detected using CCK‐8 assay (right panel). C, Blockage of AMPK inhibited colony formation. Kyse30 and Kyse450 cells were treated with PR‐619 single (3 µmol/L) or combined with CC (2 µmol/L) for 10 days and then fixed, stained captured (left panel) and counted (right panel). D, Blockage of AMPK accelerated PR‐619 triggered apoptosis. Kyse30 and Kyse450 cells were treated with PR‐619 single (8 µmol/L) or combined with CC (8 µmol/L). Apoptosis was determined by FACS analysis using Annexin V‐FITC/PI double‐staining kit (left panel), and Annexin V^+^ cell populations were defined as apoptosis (right panel). E, Effect of CC and PR‐619 co‐treatment on protein expression. Kyse30 and Kyse450 cells were treated with PR‐619 single (8 µmol/L) or combined with CC (8 µmol/L). Then, proteins were extracted and analysed by Western blotting with specific antibodies. GAPDH was used as the loading control. All data were representative of at least three independent experiments (n = 3; error bar, SD)

To further confirm the role of AMPK in PR‐619‐induced autophagy, Kyse450 and Kyse30 cells were co‐treated with PR‐619 and Compound C (CC), an AMPK inhibitor.^48^ Compared with PR‐619 alone, CC and PR‐619 co‐treatment effectively downregulated the expression of p‐AMPKα and attenuated PR‐619 induced autophagy, as demonstrated by reduced LC3 puncta‐positive cells (Figure 6A) and LC3II expression (Figure 6E). Meanwhile, AMPK inhibition significantly enhanced PR‐619‐induced cell growth inhibition (Figure 6B‐C) and cell apoptosis (Figure 6D‐E). These results implicate that Ca^2+^‐CaMKKβ‐AMPK signalling possibly plays a crucial role in PR619‐induced autophagy. AMPK inhibition enhanced PR‐619‐induced cytotoxicity, probably through blocking autophagy and facilitating cell apoptosis.

### 3.7 Accumulation of poly‐ubiquitinated proteins was responsible for the effect of PR‐619

**FIGURE 7:**
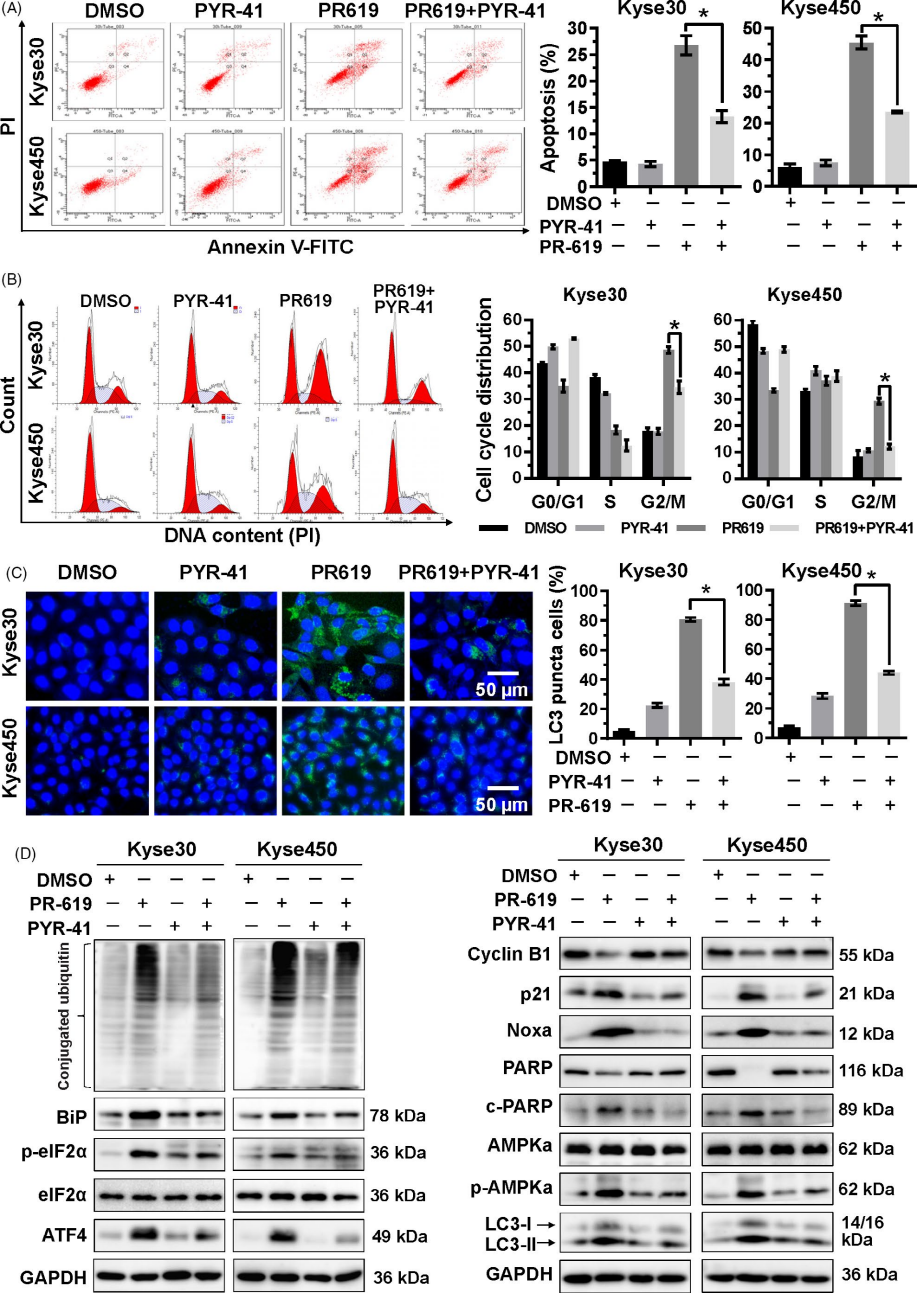
Accumulation of ubiquitinated proteins participated in the cell growth inhibition effect of PR‐619. A, PYR‐41, ubiquitin E1 inhibitor, increased PR‐619 induced apoptosis. Kyse30 and Kyse450 cells were treated with PR‐619 (8 µmol/L) single or combined with PYR‐41 (20 µmol/L). Apoptosis was determined by FACS analysis using Annexin V‐FITC/PI double‐staining kit (left panel) and Annexin V^+^ cell populations were defined as apoptosis (right panel). B, PYR‐41 decreased G2/M cell cycle arrest. Kyse30 and Kyse450 cells were treated with PR‐619 single (8 µmol/L) or combined with PYR‐41 (20 µmol/L), followed by PI staining and FACS analysis for cell cycle profile (left panel). Distribution was analysed by Modifit and GraphPad software (right panel). C, PYR‐41 reduced PR‐619 induced autophagy. Kyse30 and Kyse450 cells were treated with PR‐619 single (8 µmol/L) or combined with PYR‐41 (20 µmol/L). LC3 was detected using immunofluorescence assay, reprehensive pictures were captured (left panel), and LC3 puncta cells were statistically analysed (right panel). D, Effect of PYR‐41 on protein expression of ER stress, cell cycle‐related proteins, Noxa, c‐PARP, PARP, p‐AMPKα, AMPKα and LC3. Kyse30 and Kyse450 cells were treated with PR‐619 single (8 µmol/L) or combined with PYR‐41 (20 µmol/L). Cell proteins were detected using specific antibodies. GAPDH was used as the loading control. All data were representative of at least three independent experiments (n = 3; error bar, SD)

PR‐619 treatment accumulated the poly‐ubiquitinated proteins (Figure 3F). To examine whether accumulation of poly‐ubiquitinated proteins was responsible for the effect of PR‐619, ubiquitin E1 inhibitor, PYR‐41, was used to diminish the accumulation of poly‐ubiquitinated proteins in PR‐619‐treated cells. Results showed that PYR‐41 treatment decreased apoptosis (Figure 7A), G2/M cell cycle arrest (Figure 7B) and autophagy (Figure 7C) in PR‐619‐treated cells. PYR‐41 effectively diminished the accumulation of conjugated ubiquitin and inhibited ER stress that induced by PR‐619 (Figure 7D). Following the above results (Figures 1D, 3E and 5E), PYR‐41 downregulated the expression of ATF4‐Noxa and the cleavage of PARP; rescued the expression of Cyclin B1 and downregulated the protein level of p21; and decreased the expression of phosphorylated AMPKα and LC3II (Figure 7D) in PR‐619‐treated cells. Collectively, these findings suggested that the accumulation of poly‐ubiquitinated proteins induced ER stress and was involved in the effect of PR‐619.

## 4 DISCUSSION

Low early diagnosis rate and drug resistance are the main reasons for the high death rate of ESCC. It is crucial to study the mechanistic basis for ESCC progression and develop new therapeutic strategies fighting ESCC. In previous studies, we demonstrated that inactivation of USP7 or USP14 with specific inhibitor led to the effective suppression of ESCC cell growth in vitro and in murine model.^13^, ^15^ However, it is unknown whether broad‐range DUB inhibitor PR‐619 could also significantly inhibit ESCC cell growth via disrupting the dynamic balance of ubiquitin conjugation. Here, our results showed that PR‐619 treatment induced the accumulation of ubiquitinated proteins and led to effective suppression of ESCC cell growth by inducing G2/M cell cycle arrest and apoptosis. Meanwhile, PR‐619 treatment could trigger protective autophagy. Blocking autophagy with CQ or BafA1 could further enhance the growth inhibition efficacy of PR‐619 on ESCC cells.

PR‐619 is a general inhibitor of DUBs, including USP22 and USP14.^25^ Previous studies showed that USP22 and USP14 worked as deubiquitinase of cyclin B1 and stabilized cyclin B1 during the G2/M phase.^49^, ^50^ Knocking‐down of USP14 arrested cell cycle at G2/M phase.^50^ Per these reports, we found that PR‐619 treatment led to G2/M cell cycle arrest accompanied by the downregulated expression of cyclin B1. However, Ling *et al* reported that knockdown of USP22 by siRNA induced cells G0/G1 cell cycle arrest via the c‐Myc/cyclin D2 pathway in HepG2 cells.^51^ Most recently, Kuo *et al* revealed that PR‐619 treatment induced G0/G1 cell cycle arrest in bladder urothelial carcinoma cells with decreased p‐Histone H3 (Ser10) and increased p21 and phospho‐CDK2 (Tyr15).^34^ Our results showed that PR‐619 treatment also decreased the expression of p‐Histone H3 (Ser10) and increased the expression of p21. Nevertheless, PR‐619 treatment had little effect on the expression of c‐Myc. Besides, ubiquitin E1 inhibitor PYR‐41 rescued the expression of Cyclin B1, downregulated p21 and reduced the accumulation of G2/M phase cells in PR‐619‐treated ESCC cells, which implied that PR‐619 triggered G2/M cell cycle arrest in ESCC by regulating the expression of cyclin B1 and p21.

Accumulating evidence shows that ER stress is a vital mechanism response to DUB inhibition.^15^, ^52^, ^53^ Noxa is involved in ER stress‐induced cell death.^43^, ^54^ Meanwhile, eIF2α‐ATF4 plays an essential role in Noxa activation and Noxa‐mediated apoptosis.^35^, ^54^ In the present study, we found that PR‐619 treatment led to the accumulation of poly‐ubiquitinated proteins and triggered ER stress in ESCC cells, as evidenced by the increased expression of Bip, p‐eIF2α and ATF4. Moreover, we found that PR‐619 treatment led to Noxa‐mediated apoptosis, which was dependent on ATF4. PR‐619 treatment increased the protein level of ATF4 in a dose‐dependent manner; downregulation of ATF4 via siRNA effectively blocked the expression of Noxa and apoptosis. Furthermore, ubiquitin E1 inhibitor PYR‐41 could alleviate the accumulation of conjugated ubiquitin, inhibit ER stress, decrease the expression of ATF4‐Noxa and cleaved PARP in PR‐619‐treated cells. Collectively, these findings suggest that the accumulation of ubiquitinated proteins is involved in the anti‐cancer mechanism of PR‐619. ATF4‐Noxa is partially responsible for the cell growth inhibition of oesophageal cancer by PR‐619 treatment.

It is known that ER stress can disrupt Ca^2+^ homeostasis, induce the influx of Ca^2+^ leakage into intracellular cytosol^45^, ^55^ and thus stimulate macroautophagy by CaMKKβ‐AMPK signalling pathway.^45^, ^47^, ^56^ Multiple lines of evidence indicate that inhibition of the 26S proteasome and accumulation of ubiquitinated proteins induce protective autophagy through an AMPK‐dependent pathway.^57^, ^58^ Compound C treatment could significantly block AMPK activation and downregulate the level of phosphorylated AMPK (Thr172) and autophagy, evidenced by decreased levels of LC3‐II.^59^ Deshmukh *et al* reported that a variety of proteasome inhibitors activates AMPK primarily mediated by Calcium/CaMKKβ.^60^ Recently, Seiberlich *et al* reported that PR‐619 treatment led to the activation of autophagy.^32^ However, the underlying mechanisms need to be investigated. Here, we demonstrated that PR‐619 treatment triggered ER stress, increased intracellular Ca^2+^ concentration and induced protective autophagy in oesophageal cancer cells. PYR‐41 could reduce the accumulation of ubiquitinated proteins, alleviate ER stress and decrease autophagy in PR‐619‐treated cells. Besides, the treatment of STO‐609, a CaMKKβ inhibitor, led to the inactivation of AMPKα induced by PR‐619, decreased the expression of p‐AMPKα and accompanied by reduced LC3‐II. Furthermore, treatment with AMPK inhibitor CC could effectively inhibit PR‐619‐induced autophagy and enhanced apoptosis. These results implied that Ca^2+^‐CaMKKβ‐AMPK played an essential role in PR‐619‐induced autophagy in oesophageal cancer cells.

**FIGURE 8:**
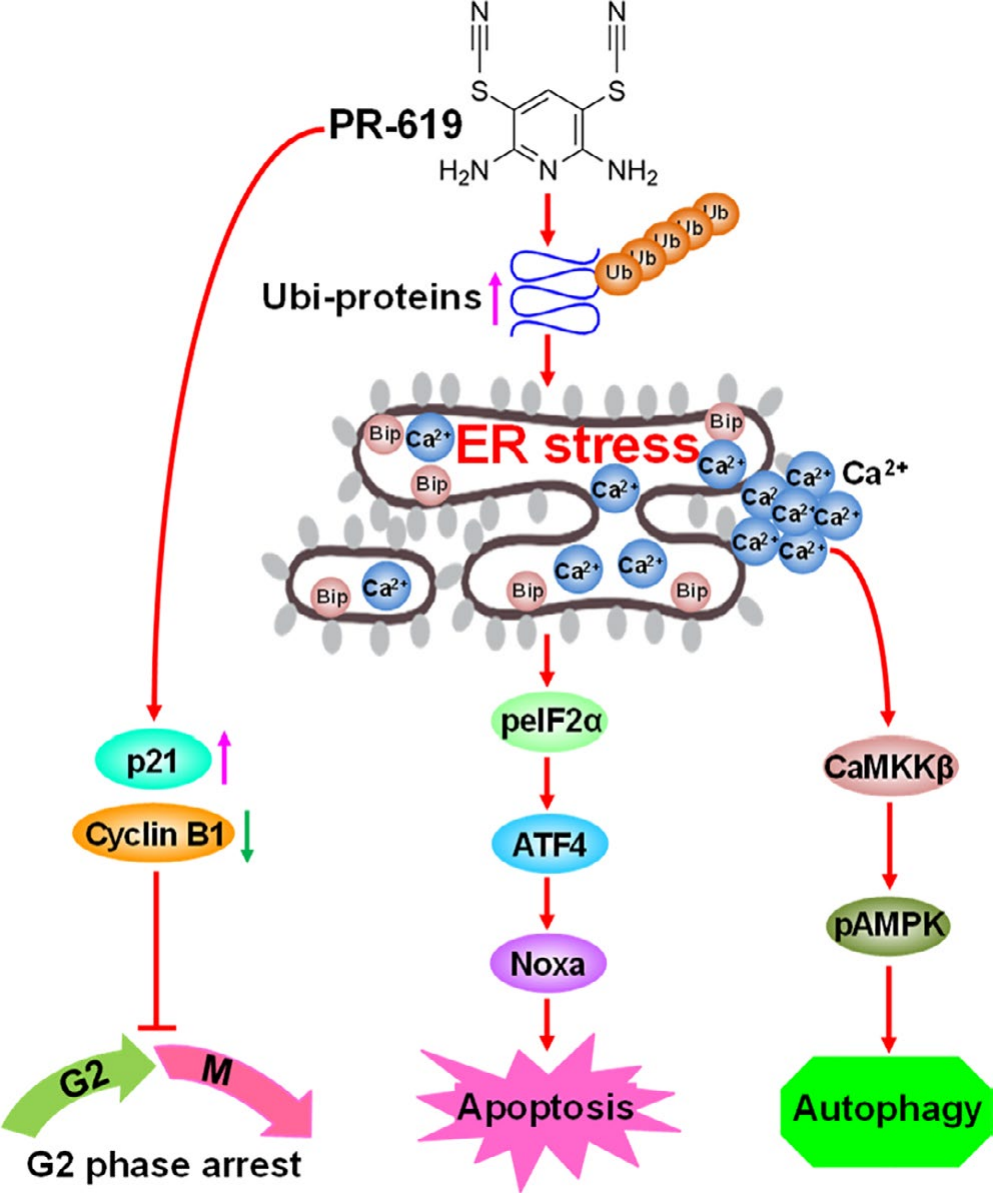
Schema of the mechanism for PR‐619‐induced cell cycle arrest, apoptosis and autophagy in ESCC

Collectively, this study revealed the detailed mechanism of PR‐619 in ESCC cells (Figure 8). PR‐619 treatment led to G2/M cell cycle arrest by downregulating the expression of cyclin B1 and upregulating the protein level of p21. Meanwhile, PR‐619 treatment induced the accumulation of ubiquitinated proteins induced ER stress and triggered ATF4‐Noxa‐mediated apoptosis. Besides, PR‐619 could activate autophagy through Ca^2+^‐CaMKKβ‐AMPK signalling. Our findings provide not only new insight into the cytotoxic action of PR‐619 in ESCC but also trigger a strong impetus for the clinical investigation of PR‐619 for the treatment of ESCC.

## CONFLICT OF INTEREST

The authors declare that there are no conflicts of interest.

## AUTHOR CONTRIBUTIONS

T. H. and P. C. involved in conception and design. L. W., M. L., B. S., X. H., Y. S., M. Z., Y. X., P. L., Y. W., Y. G. and J. L. involved in performing the experiments. L. W., M. L., T. H. and J. S. involved in analysis and interpretation of data. T. H. and P. C. contributed to the writing. P. L. and J. S. contributed to the review and revision of the manuscript. All authors have reviewed and approved the final version of the manuscript.

## ETHICAL STATEMENT

Not applicable.

## Data Availability

The data that support the findings of this study are available from the corresponding author upon reasonable request.
